# Epidemiology of Acute Symptomatic Seizures among Adult Medical Admissions

**DOI:** 10.1155/2016/4718372

**Published:** 2016-01-24

**Authors:** Paul Osemeke Nwani, Maduaburochukwu Cosmas Nwosu, Monica Nonyelum Nwosu

**Affiliations:** ^1^Clinical Pharmacology and Therapeutics Unit/Neurology Unit, Department of Medicine, Nnamdi Azikiwe University Teaching Hospital, PMB 5025, Nnewi 435101, Anambra State, Nigeria; ^2^Neurology Unit, Department of Medicine, Nnamdi Azikiwe University Teaching Hospital, PMB 5025, Nnewi 435101, Anambra State, Nigeria; ^3^Gastroenterology Unit, Department of Medicine, Nnamdi Azikiwe University Teaching Hospital, PMB 5025, Nnewi 435101, Anambra State, Nigeria

## Abstract

Acute symptomatic seizures are seizures occurring in close temporal relationship with an acute central nervous system (CNS) insult. The objective of the study was to determine the frequency of presentation and etiological risk factors of acute symptomatic seizures among adult medical admissions. It was a two-year retrospective study of the medical files of adults patients admitted with acute symptomatic seizures as the first presenting event. There were 94 cases of acute symptomatic seizures accounting for 5.2% (95% CI: 4.17–6.23) of the 1,802 medical admissions during the period under review. There were 49 (52.1%) males and 45 (47.9%) females aged between 18 years and 84 years. The etiological risk factors of acute symptomatic seizures were infections in 36.2% (*n* = 34) of cases, stroke in 29.8% (*n* = 28), metabolic in 12.8% (*n* = 12), toxic in 10.6% (*n* = 10), and other causes in 10.6% (*n* = 10). Infective causes were more among those below fifty years while stroke was more in those aged fifty years and above. CNS infections and stroke were the prominent causes of acute symptomatic seizures. This is an evidence of the “double tragedy” facing developing countries, the unresolved threat of infectious diseases on one hand and the increasing impact of noncommunicable diseases on the other one.

## 1. Introduction

Acute symptomatic seizures are clinical seizures occurring in close temporal relationship with an acute central nervous system (CNS) insult, which may be metabolic, toxic, structural, infectious, or inflammatory [1]. Such seizures are considered to be an acute manifestation of the insult and may not recur when the underlying cause has been removed or the acute phase has elapsed [2].

Acute symptomatic seizures represent about 40% of all cases of afebrile seizures in developed countries and more than half in some geographic areas, for example, where cysticercosis is endemic [2, 3]. Acute symptomatic seizures are more frequent among males and in the extremes of age (youngest age class and in the elderly) [2]. The causes of acute symptomatic seizures in developed countries may differ from the causes in developing countries [4]. The commonest causes among adults in developed nations include traumatic brain injury, stroke, medication, or alcohol withdrawal, brain tumors, and metabolic insult [2, 5].

Treatment of acute symptomatic seizures requires the simultaneous treatment of the underlying aetiology and the use of anticonvulsant drugs [6]. The anticonvulsants preferred for the treatment of acute symptomatic seizures are those available for intravenous use, such as benzodiazepines, fosphenytoin or phenytoin, valproate, levetiracetam, and phenobarbital [6]. Acute symptomatic seizures are an undisputable risk factor for epilepsy [2].

When seizures complicate acute neurological disorders, they add an additional layer of complexity to patient management [4]. The knowledge of the etiologic risk factors of acute symptomatic seizures in third-world countries will invariably contribute to the effort aimed at preventing and managing medical conditions frequently complicated by seizures. Currently there is dearth of information on the epidemiology of acute symptomatic seizures among adult medical admissions in Nigeria and Africa in general.

## 2. Methods

This was a retrospective study of medical records of adults patients admitted with acute symptomatic seizures as the first presenting event at medical wards of the Nnamdi Azikiwe University Teaching Hospital (NAUTH), Nnewi. NAUTH is the largest medical referral centre in Anambra State, Southeast Nigeria. Anambra State occupies an area of 4,844 Km sq. and has a population of 4,182,032 according to the 2006 Nigeria population census. The average attendance of NAUTH has been on the increase since inception of the hospital and currently the average annual attendance is 117,351 patients (103,601 outpatients and 13,750 inpatients) accounting for a 2.8% medical coverage of population of over four million of Anambra State. The current annual adult medical admissions of NAUTH are about 1,992 accounting for 14.5% (*n* = 1,992/13,750) of the entire hospital admissions. Patients from all clinical subspecialties in internal medicine are admitted to the two medical wards of the hospital either from the accident and emergency department or from the medical outpatient clinics.

The medical records of all medical wards admissions from January 2005 to December 2006 were retrieved from the records department of the hospital and reviewed. Those who presented with seizures as a presenting complaint were selected and analysed. Data extracted from the medical record files included demographic data (age and sex), relevant history and clinical examination findings, available investigation results, and diagnosis. All cases of acute symptomatic seizures are reviewed by the neurologists in our centre.

Acute symptomatic seizure was defined as a clinical seizure occurring in close temporal relationship with an acute central nervous system (CNS) insult, which may be metabolic, toxic, structural, infectious, or inflammatory [1].

Seizures were defined as acute symptomatic ones if they occurred within one week of stroke, central nervous system infection, or systemic infection or if they occur in the presence of severe metabolic derangements documented by biochemical abnormalities obtained within the immediate period of the metabolic event. The definitions used for the various etiologic agents are as follows.

Seizures following stroke were defined as acute symptomatic ones if they occurred within seven days of stroke. Stroke was defined as sudden onset focal or global neurological deficit of vascular origin lasting for more than 24 hours or resulting in death. Stroke type was categorized using the World Health Organisation stroke criteria and/or brain CT scan.

Seizures occurring in relation to meningoencephalitis and sepsis in our study were defined as acute symptomatic ones if they occurred within seven days of the events. The diagnosis of meningoencephalitis was based on documentation of fever, headache, alteration in the level of consciousness and signs of meningism on examination, and a positive cerebrospinal fluid result with or without isolation of pathogen. Sepsis was diagnosed based on documented clinical and laboratory (hematologic indices) evidence of systemic infection in a patient who does not meet the criteria of meningoencephalitis as defined above.

Metabolic causes of seizures were diagnosed based on documentation of appropriate laboratory results obtained within the periods of the seizures and other relevant clinical data. Uremic encephalopathy was diagnosed as cause of seizure based on documentation of azotemia within the periods of the seizures in a patient with relevant clinical history and findings. Hepatic encephalopathy was diagnosed as cause of acute symptomatic seizures if seizures occurred during the period of overt neuropsychiatric symptoms in a patient with history of liver disease.

Acute symptomatic seizures associated with hypertensive encephalopathy were defined as seizures occurring in patients with severe elevation of blood pressure, altered mental status, or evidence of diffuse brain dysfunction with prompt response to antihypertensive therapy.

The cases of acute symptomatic seizures relating to alcohol use were seizures occurring in patients with history of chronic alcohol abuse, presence of alcohol withdrawal symptoms, and seizures within 48 hours of last drink.

The diagnosis of brain space occupying lesions for cases with HIV/AIDS was based on clinical history, examination findings, and/or brain CT scan.

Eleven patients had brain CT scan; of these patents ten were cases of acute symptomatic seizures due to stroke while one was due to HIV infection.

Patients with epilepsy and those below 18 years of age were excluded (patients below 18 years are admitted to the hospital paediatric unit).

*Statistical Analysis*. Data collected was analysed using Statistical Package for the Social Sciences SPSS version 15 (SPSS Inc., Chicago, IL, USA). Prevalence values with their 95% confidence intervals (95% CI) were calculated. Relevant percentages, frequencies, means and standard deviations, and confidence intervals were calculated. Findings were represented with tables and a figure.

## 3. Results

There were 94 cases of acute symptomatic seizures accounting for 5.2% (95% CI: 4.17–6.23) of the 1,802 medical admissions during the period under review. There were 49 (52.1%) males and females 45 (47.9%) but the observed difference was not statistically significant *χ* ^2^ = 7.063, *p* = 0.422. The patients were aged between 18 and 84 years with a mean age of 51 ± 16 years (males: 49.8 ± 16; females: 53.7 ± 15.7).

The etiological risk factors of acute symptomatic seizures were infections in 36.2% (*n* = 34) of cases, stroke in 29.8% (*n* = 28), metabolic in 12.8% (*n* = 12), and toxic in 10.6% (*n* = 10) (Table 1). The 28 cases of stroke accounted for 24.3% (*n* = 28/115) of all stroke admissions during the period under review while the 34 infectious cases accounted for 18.9% (*n* = 34/201) of cases of infectious diseases admitted during the period.

The group designated “uncertain” did not have sufficient data to be classified into any of the groups as defined above and where indicated as patients living with epilepsy. Two cases of seizures due to brain tumours that presented during the period under review were excluded as acute symptomatic ones since they belong to the group progressive symptomatic according to the proposed ILEA definition as they occur in the context of an evolving clinical condition [1]. There were no documented cases of acute symptomatic seizures caused by inflammation during the period under review.

**Figure 1:**
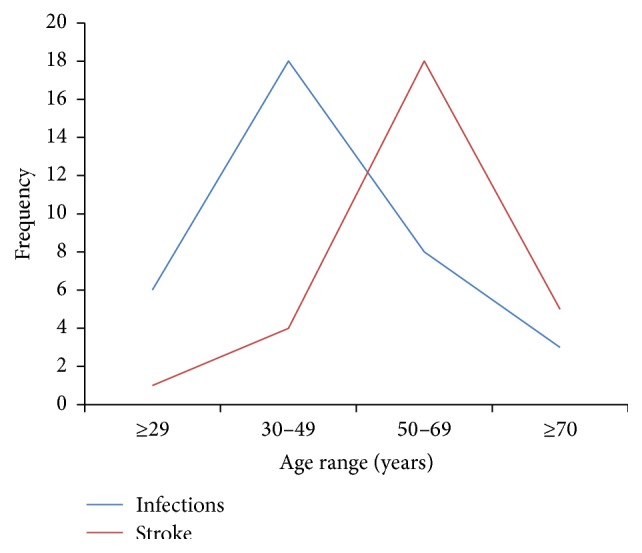
Age distribution of the two major causes of acute symptomatic seizures.

Figure 1 shows the relative age distribution of the two major etiological risk factors. Infectious causes peaked at 30–49 age group and those aged below 49 years accounted for 70.6% (*n* = 24/34) of seizures due to infectious causes while stroke peaked at 50–69 age group and those aged 50 years and above accounted for 82.1% (*n* = 23/28) of cases of seizures due to stroke.

## 4. Discussion

### 4.1. Prevalence of Acute Symptomatic Seizures

The prevalence of acute symptomatic seizures among medical admissions found in our study was 5.2% (95% CI: 4.17–6.23). This is more than 2.1% reported among neurological intensive care patients in India and 3.5% reported among medical intensive care unit patients in the USA [4, 7]. Though methodological differences and differences in patient selection limited direct comparisons of the results of these previous studies and our study, the higher prevalence found in our study may indicate a high frequency of acute symptomatic seizures among our medical admissions.

There was an insignificant male preponderance of acute symptomatic seizures in our study. The risk of acute symptomatic seizures in males is almost double that of females in population based studies [5]. This sex difference has been attributed to the incidence of underlying risk factors like head trauma rather than any biological phenomenon [5]. Our study was among medical admissions which excluded risk factors like traumatic brain injuries which are commoner in males; this may in part account for the insignificant sex difference we observed.

### 4.2. Aetiology of Acute Symptomatic Seizures

Infectious causes were the most frequent cause of acute symptomatic seizures in our study accounting for 36.2% (*n* = 34/94) of cases. This is comparable to 32% (*n* = 21/66) reported among patients admitted to a neurological intensive care unit in India [4]. Among the infective causes meningoencephalitis accounted for 13.8% (*n* = 13/94) of acute symptomatic seizures in this present study and this is comparable to the Indian study where meningoencephalitis ranked highest among the infective causes of acute symptomatic seizures. Studies in western nations report higher frequencies of etiologic risk factors like stroke, medication or alcohol withdrawal, brain tumor, and eclampsia among adults while infections are dominant causes of acute symptomatic seizures in newborns and children [5]. The risk of acute symptomatic seizures occurring in patients with acute central nervous system infections is more with encephalitis than meningitis [8, 9]. In our study, the absence of results of appropriate investigational facilities due to their unavailability or high cost in part limited further differentiation of these two entities among those with meningoencephalitis. Sepsis accounted for 7.5% (*n* = 7/94) of cases of acute symptomatic seizures in this present study. Seizures in these cases may be due to encephalopathy relating to such factors, hemodynamic dysfunction and metabolic derangement in patients with severe infections. Sepsis is a documented frequent cause of encephalopathy [7].

Human immunodeficiency virus (HIV) infection was the etiological risk factor for 11.7% (*n* = 11/94) of cases of acute symptomatic seizures in our study. Acute symptomatic seizures are common among HIV infected individuals and are present in up to 2% to 20% of cases [10]. Identifiable causes of acute symptomatic seizures in HIV infected individuals in this present study were HIV encephalopathy, meningoencephalitis, and space occupying lesions (SOL) from probable opportunistic infections. This agrees with reported causes of seizures in HIV infected persons like opportunistic infections, systemic illness, drug or alcohol abuse, antiretroviral drug usage, and acquired immunodeficiency syndrome (AIDS) encephalopathy [10].

In our study neurocysticercosis was not among the causes of acute symptomatic seizures. This may indicate a low frequency of neurocysticercosis in our environment or under diagnosis of the condition due to lack of adequate facilities to make the diagnosis. Neurocysticercosis has been rarely reported in Nigeria hospitals studies even with the reports on the high prevalence of cysticercosis in meat in various parts of the country [11–13]. In a prospective study in India, neurocysticercosis ranked the same as meningoencephalitis as infective cause of acute symptomatic seizures [4]. In places where neurocysticercosis is endemic it is the most common cause of epilepsy in developing countries, where it accounts for up to 30% of all seizures [14].

Cerebral malaria which is a common cause of acute febrile seizures in children in developing countries including Nigeria accounted for only 2.1% (*n* = 2/94) of acute seizures in our study [15]. Cerebral malaria is rare among adults in the tropics and this has been attributed to the development of immunity against malaria under the stable endemic conditions prevailing in the region [16].

Stroke was a common cause of acute symptomatic seizures in our study accounting for 29.8% (*n* = 28/94) of cases. Acute symptomatic seizures following stroke tend to occur in 2.4% to 6.3% of patients but in our study acute symptomatic seizures were present in 24.3% of cases [17, 18]. This higher frequency of acute symptomatic seizures following stroke found in our study may be related to the poor initial management of the acute stage of stroke and the attendant complications of such managements. Most stroke patients in this part of the country present initially to nonspecialist with little knowledge and inadequate facilities for acute management of stroke and are referred to specialist centres only when complications begin to develop. In a prospective multicentre study most of the seizures following stroke occurred within 2 days and almost half (43%) within 24 hours after the stroke [19]. The frequency of acute symptomatic seizures following stroke is more with haemorrhagic than ischemic stroke as was found in this present study [20].

Metabolic derangements accounted for 18.1% (*n* = 17/94) of cases of acute symptomatic seizures in this present study. Unlike the India study where hyponatremia accounted for most metabolic derangement hyperglycaemia accounting for 6.4% (*n* = 6/94) and hypoglycaemia accounting for 5.3% (*n* = 5/94) were the most frequent metabolic contributors in our study [4]. Such disparities in the metabolic causes of seizures between their study and ours may arise from differences in frequencies of the major primary diagnosis in both series. Also the level of baseline medical care available to the patients before the onset of seizures may be contributory since most of the metabolic causes in our study were due to poor glycaemic control. Hyperglycaemic emergencies are significant causes of endocrine admission and deaths in Nigeria [21]. Among hyperglycaemic comas in diabetes, seizures are more frequently encountered in hyperosmolar hyperglycaemic state than in diabetic ketoacidosis probably due to the anticonvulsant effect of ketosis [22].

In our study poisoning and alcohol related causes were implicated in 4.3% (*n* = 4/94) and 3.2% (*n* = 3/94) of cases of acute symptomatic seizures, respectively. Acute symptomatic seizures associated with alcohol intake may occur after alcohol withdrawal (as in our study) or acute intoxication and this can represent as much as one-third of total hospital admissions due to seizures [23]. The names of the implicated medications in two cases were documented as unknown in the record files but the clinical histories were highly suspicious of the medications poisoning. This highlights not too infrequent problem associated with studying drug related problems in developing countries. Due to the low level of literacy the patients have little knowledge of the drugs they are taking. This is further complicated by concealment of drug names from the patients by the prescribers in most private owned medical establishments. The concomitants use of herbal and orthodox medications even further complicates the matter and undermines adequate study of the medications use especially when they are involved in suspected cases of drug toxicity.

### 4.3. Study Limitations

This study however had several limitations owning to the retrospective nature of the study and the frequent problems of poor record keeping associated with such studies. However, notwithstanding these limitations the findings afford a baseline for further studies on this subject in developing nations. The study was among medical admissions and so did not include etiologic risk factors like traumatic brain injuries which are managed primarily by the neurosurgical teams. Cases of acute symptomatic seizures developing in patients admitted without seizures as the first presenting feature were also not captured because of problems of inadequate documentations not infrequent with retrospective studies like this present study. This study was also limited by unavailability of high yield neurologic investigative modalities (like serological test, electroencephalography, viral studies, neuroimaging, and others) that would have enhanced accurate delineation of the differential diagnosis of the causes of acute symptomatic seizures. This is because of the absence of such facilities and for those that are available their high cost limits their use since patients pay out of pocket for medical facilities in the countries.

## 5. Conclusion

Infections and stroke were the prominent causes of acute symptomatic seizures. This an evidence of the “double tragedy” facing developing countries, the unresolved threat of infectious diseases on one hand and the increasing impact of noncommunicable diseases on the other one. The need for further studies in this area has been made bare given the high frequency of this condition found in this study.

## Figures and Tables

**Table 1:** Classes of etiological risk factors of acute symptomatic seizures.

Disease class	Frequency (*n* = 94)	Diseases	Frequency (*n* = 94)	Percentage (%)
Infectious	34 (36.2%)	Meningoencephalitis	13	13.8
		HIV/AIDS^*∗*^	11	11.7
		Sepsis	7	7.4
		Cerebral malaria	2	2.1
		Cerebral abscess	1	1.1

Stroke	28 (29.8%)	Haemorrhagic stroke	14	14.9
		Ischaemic stroke	9	9.6
		Subarachnoid haemorrhage	5	5.3

Metabolic	17 (18.1%)	Hyperglycaemia	6	6.4
		Hypoglycaemia	5	5.3
		Uraemia	3	3.2
		Hepatic	2	2.1
		Hyponatremia	1	1.1

Toxic	7 (7.4%)	Poisoning^*∗∗*^	4	4.3
		Alcohol	3	3.2

Others	8 (8.5%)	Hypertensive encephalopathy	5	5.3
		Uncertain	3	3.2

^*∗*^HIV/AIDS (encephalopathy: 5, space occupying lesion: 4, and meningoencephalitis: 3), ^*∗∗*^poisoning (CO poisoning: 1, organophosphate: 1, and unknown medication: 2).

## References

[1] Beghi E., Carpio A., Forsgren L. (2010). Recommendation for a definition of acute symptomatic seizure. *Epilepsia*.

[2] Hauser W. A., Beghi E. (2008). First seizure definitions and worldwide incidence and mortality. *Epilepsia*.

[3] Pal D. K., Carpio A., Sander J. W. A. S. (2000). Neurocysticercosis and epilepsy in developing countries. *Journal of Neurology Neurosurgery and Psychiatry*.

[4] Narayanan J., Murthy J. M. K. (2007). New-onset acute symptomatic seizure in a neurological intensive care unit. *Neurology India*.

[5] Annegers J. F., Hauser W. A., Lee J. R.-J., Rocca W. A. (1995). Incidence of acute symptomatic seizures in Rochester, Minnesota, 1935–1984. *Epilepsia*.

[6] Koppel B. S. (2009). Treatment of acute and remote symptomatic seizures. *Current Treatment Options in Neurology*.

[7] Bleck T. P., Smith M. C., Pierre-Louis S. J.-C., Jares J. J., Murray J., Hansen C. A. (1993). Neurologic complications of critical medical illnesses. *Critical Care Medicine*.

[8] Annegers J. F., Hauser W. A., Beghi E., Nicolosi A., Kurland L. T. (1988). The risk of unprovoked seizures after encephalitis and meningitis. *Neurology*.

[9] Kim M. A., Park K. M., Kim S. E., Oh M. K. (2008). Acute symptomatic seizures in CNS infection. *European Journal of Neurology*.

[10] Dal Pan G. J., McArther J. C., Harrison M. J. G., Berger J. R., Levy R. M. (1997). Neurological symptoms in HIV infection. *AIDS and Nervous System*.

[11] Ogunrin A. O. (2006). Epilepsy in Nigeria—a review of etiology, epidemiology and management. *Benin Journal of Postgraduate Medicine*.

[12] Weka R. P., Ikeh E., Kamani J. (2013). Seroprevalence of antibodies (IgG) to *Taenia solium* among pig rearers and associated risk factors in Jos metropolis, Nigeria. *Journal of Infection in Developing Countries*.

[13] Usip L. P. E., Isaac L., Amadi E. C., Utah E., Akpaudo U. (2011). The occurrence of cysticercosis in cattle and Taeniasis in man in Uyo, capital city of Akwa Ibom State, Nigeria. *Nigerian Journal of Agriculture, Food and Environment*.

[14] Medina M. T., Rosas E., Rubio-Donnadieu F., Sotelo J. (1990). Neurocysticercosis as the main cause of late-onset epilepsy in Mexico. *Archives of Internal Medicine*.

[15] Oluwayemi I. O., Brown B. J., Oyedeji O. A., Oluwayemi M. A. (2013). Neurological sequelae in survivors of cerebral malaria. *Pan African Medical Journal*.

[16] Dondorp A. M. (2005). Pathophysiology, clinical presentation and treatment of cerebral malaria. *Neurology Asia*.

[17] Lamy C., Domigo V., Semah F. (2003). Early and late seizures after cryptogenic ischemic stroke in young adults. *Neurology*.

[18] Beghi E., D'Alessandro R., Beretta S. (2011). Incidence and predictors of acute symptomatic seizures after stroke. *Neurology*.

[19] Bladin C. F., Alexandrov A. V., Bellavance A. (2000). Seizures after stroke: a prospective multicenter study. *Archives of Neurology*.

[20] Labovitz D. L., Hauser W. A., Sacco R. L. (2001). Prevalence and predictors of early seizure and status epilepticus after first stroke. *Neurology*.

[21] Ogbera A. O., Chinenye S., Onyekwere A., Fasanmade O. (2007). Prognostic indices of diabetes mortality. *Ethnicity and Disease*.

[22] Gao X., Wee A. S., Nick T. G. (2005). Effect of keto-acidosis on seizure occurrence in diabetic patients. *Journal of the Mississippi State Medical Association*.

[23] Earnest M. P., Yarnell P. R. (1976). Seizure admissions to a city hospital: the role of alcohol. *Epilepsia*.

